# One day of environment-induced heat stress damages the murine myocardium

**DOI:** 10.1152/ajpheart.00180.2024

**Published:** 2024-08-30

**Authors:** Melissa Roths, Tori E. Rudolph, Swathy Krishna, Alyona Michael, Joshua T. Selsby

**Affiliations:** ^1^Department of Animal Science, Iowa State University, Ames, Iowa, United States; ^2^Veterinary Diagnostic Laboratory, Iowa State University College of Veterinary Medicine, Ames, Iowa, United States

**Keywords:** climate change, heat stroke, muscle, thermic injury

## Abstract

The physiological consequences of environment-induced heat stress (EIHS), caused by prolonged exposure to excess heat and humidity, are largely unknown. The purpose of this investigation was to determine the extent to which EIHS alters cardiac health. We hypothesized that 24 h of EIHS would cause cardiac injury and cellular dysfunction in a murine EIHS model. To test this hypothesis, 7-wk-old female mice were housed under thermoneutral (TN) conditions (*n* = 12; 31.2 ± 1.01°C, 35 ± 0.7% humidity) or EIHS conditions (*n* = 14; 37.6 ± 0.01°C, 42.0 ± 0.06% humidity) for 24 h. Environment-induced heat stress increased rectal temperature by 2.1°C (*P* < 0.01) and increased subcutaneous temperature by 1.8°C (*P* < 0.01). Body weight was decreased by 10% (*P* = 0.03), heart weight/body weight was increased by 26% (*P* < 0.01), and tissue water content was increased by 11% (*P* < 0.05) in EIHS compared with TN. In comparison with TN, EIHS increased protein abundance of heat shock protein (HSP) 27 by 84% (*P* = 0.01); however, HSPs 90, 60, 70, and phosphorylated HSP 27 were similar between groups. Histological inspection of the heart revealed that EIHS animals had increased myocyte vacuolation in the left ventricle (*P* = 0.01), right ventricle (*P* < 0.01), and septum (*P* = 0.01) compared with TN animals. Biochemical indices are suggestive of mitochondrial remodeling, increased autophagic flux, and robust activation of endoplasmic reticulum stress in hearts from EIHS mice compared with TN mice. These data demonstrate that 1 day of EIHS is sufficient to induce myocardial injury and biochemical dysregulation.

**NEW & NOTEWORTHY** The consequences of prolonged environment-induced heat stress (EIHS) on heart health are largely unknown. We discovered that a 24-h exposure to environmental conditions sufficient to cause EIHS resulted in cardiac edema and histopathologic changes in the right and left ventricles. Furthermore, among other biochemical changes, EIHS increased autophagic flux and caused endoplasmic reticulum stress. These data raise the possibility that thermic injury, even when insufficient to cause heat stroke, can damage the myocardium.

## INTRODUCTION

The increased intensity, duration, and frequency of hot environmental conditions make environment-induced heat stress (EIHS) more likely. Currently, in the United States, thermic injuries result in ∼100,000 emergency room treatments annually (1), and while heat stroke can result in death, EIHS affects far more people than heat stroke and represents more than 70% of heat-related hospitalizations and emergencies (2, 3). The risk of thermic injury is not equal as outdoor workers, the elderly, people without the logistical or economic means to support artificial cooling, and individuals with impaired thermoregulation or cardiovascular function are at higher risk than others (4–6), and this may be compounded by the effects of an urban heat island (7).

The consequences of heat stroke on the cardiovascular system are well known and include arrhythmias, conduction disturbances, and myocardial ischemia/injury (8–13). In extreme cases, heat stroke can lead to heart failure, circulatory collapse, and death (10, 13–16). Even after recovery from heat stroke, victims are at a higher risk of cardiovascular disease (17–19). However, the effects of EIHS on the cardiovascular system are largely unknown, despite its far more frequent occurrence, though EIHS appears to increase the risk of future cardiovascular events and chronic kidney disease (17).

Although cardiovascular responses to EIHS have been reported in humans (20–24), they are largely limited to mild heating protocols and brief durations that are unlikely to accurately model a natural EIHS event. Using short durations of EIHS with limited thermal load in human studies is an ethical requirement; however, it prevents an understanding of the pathological consequences of EIHS and does not permit tissue collection. Likewise, previous use of rodent hyperthermia models was generally geared toward heat stroke (25–28). Using a porcine model of EIHS, we have previously demonstrated that persistent heating, using environmental conditions unlikely to cause heat stroke, leads to architectural changes and cellular dysfunction in the heart (29).

Given the increasing frequency of EIHS and the large gap in knowledge regarding EIHS-mediated impacts on the myocardium, the purpose of this investigation was to determine the extent to which EIHS alters cardiac cell health. We hypothesized that EIHS would cause myocardial injury and cellular dysfunction.

## MATERIALS AND METHODS

### Animal Treatment and Experimental Design

All animal work was approved by the Institutional Animal Care and Use Committee (IACUC-20-134) at Iowa State University. Twenty-six C57BL6 female mice (4 wk of age) were housed in groups of three in rodent Innocage bottoms prebedded with Alpha-dri, with one-ply Innorichment (M-BTM-ADE-1, Innovive IVC Rodent Caging System) with ventilated IVC single filter lids (MVX1, Innovive IVC Rodent Caging System). Mice were selected for these experiments as they are ideally suited for mechanistic investigation, whereas our pig model is well suited for translational experimentation. During acclimation, mice were housed under thermoneutral (TN) conditions and had ad libitum access to food (Prolab RMH 1000, LabDiet) and water. After 1 wk of acclimation, mice were briefly sedated with isoflurane (5% induction), a United Information Devices temperature programmable microchip (UCT-2112) implanted between the shoulder blades, and mice returned to their cages for 3 wk. At 7 wk of age, mice were moved to individual cages for 3 days and randomly assigned to either TN conditions or EIHS conditions. For environmental treatments, mice were maintained in their individual cages under TN conditions (*n* = 12; 31.2 ± 1.01°C, 35 ± 0.7% relative humidity), or to induce EIHS, the cages with conscious, unrestrained mice were placed in an environmental chamber (Avantor, Cat. No. 76205-438) for 24 h (*n* = 14; 37.6 ± 0.01°C, 42.0 ± 0.06% relative humidity). These environmental conditions were selected as preliminary experiments indicated that these conditions were likely to cause an approximate 2°C increase in rectal temperature and they are reasonable approximations of environmental conditions that may occur during naturally occurring heat events (30). All mice had free access to food and water over the duration of the environmental challenge. To allow calculation of autophagic flux, four mice/group were randomly selected from the designated TN and EIHS groups and given an intraperitoneal injection of 0.4 mg/kg colchicine 48 and 24 h before euthanasia. During environmental treatments, subcutaneous temperature (SqT) was recorded every 4 h and rectal temperature (RT) was recorded every 12 h using a rectal probe (Physitemp TH-8 Thermalert, Cat. No. 2168). After the environmental treatment, all mice were euthanized via an intraperitoneal injection of 0.25 mL pentobarbital. Hearts were removed, weighed, and cut sagittally so a portion of the heart was either fixed in 4% paraformaldehyde or frozen in liquid nitrogen for subsequent analyses.

### Protein Isolation

Whole homogenate and nuclear protein were isolated, as previously described (29, 31, 32). For whole homogenate, 20 mg of powdered heart was homogenized in a 1:10 weight-to-volume ratio in whole muscle extraction buffer (10 mM sodium phosphate buffer, pH 7.0, 2% SDS, 1% Halt protease and phosphatase inhibitor single-use cocktail) and centrifuged (10,621 *g* for 15 min at 4°C). After centrifugation, the supernatant was collected and total protein concentrations were determined using the Pierce BCA Protein Assay Kit (Cat. No. 23227 Thermo Fisher Scientific) per manufacturer’s instructions. A nuclear protein fraction (NF) was separated from the cytoplasmic fraction using NE-PER Nuclear and Cytoplasmic Extraction Reagents (Cat. No. 78833, Thermo Fisher Scientific) according to manufacturer’s instructions.

### Western Blot Analysis

Samples were prepared for Western blot analysis, as previously described (29, 31–33). Briefly, samples were diluted in 4× lithium dodecyl sulfate, denatured (85°C for 5 min), and 7 µL of each sample (28 µg whole homogenate, 7 µg NF) were loaded onto gels, separated by electrophoresis (45 min at 180 V), and transferred to nitrocellulose membranes (100 V for 1 h at 4°C). Membranes were incubated with Ponceau-S stain and the total optical density of each lane was quantified to verify equal loading using an Azure Biosystems c600 imaging system. Membranes were then blocked in 5% dehydrated, nonfat milk in TTBS (tris-buffered saline with 0.1% Tween-20) and probed with primary antibodies (Table 1) overnight at 4°C. Membranes were washed three times for 10 min in TTBS and incubated for 1 h with their respective secondary antibody at room temperature. Membranes were washed again three times for 10 min in TTBS, rocked in ECL for 5 min, and proteins were detected using an Azure Biosystems c600 imaging system. Bands were quantified using automated band detection in the AzureSpot Software (Azure, Dublin, CA).

**Table 1.** Antibodies and dilutions used for Western blot analysis

Antibody	Company/Product No.	Primary Dilution	Secondary Dilution
Adenosine monophosphate-activated kinase (AMPKα)	Cell Signaling Technology (CST), No. 5882	1:1,000	1:2,000
Phospho-AMPKα (Thr172)	CST, No. 2523	1:1,000	1:2,000
ATF4 (E4Q4E)	CST, No. 97038 mouse	1:500	1:1,000
ATF6 (D4Z8V)	CST, No. 65880	1:500	1:1,000
ATG3	CST, No. 3415	1:1,000	1:2,000
ATG7 (D12B11)	CST, No. 8558	1:1,000	1:2,000
ATG12 (D88H11)	CST, No. 4180	1:1,000	1:2,000
ATG16L1 (D6D5)	CST, No. 8089	1:1,000	1:2,000
Beclin-1(C50B12)	CST, No. 3495	1:1,000	1:2,000
BiP (C50B12)	CST, No. 3177	1:1,000	1:2,000
BCL2/adenovirus E1B 19 kDa protein-interacting protein 3-like (BNIP3L/Nix)	CST, No. 12396	1:500	1:1,000
CHOP (D46F1)	CST, No. 5554	1:500	1:1,000
Phopho-eIF2α (S51)(119A11)	CST, No. 3597	1:500	1:1,000
Eukaryotic translation initiation factor 4E-binding protein 1 (4EBP1) (53H11)	CST, No. 9644	1:6,000	1:6,000
Phospho-4EBP1 (T37746) (23604)	CST, No. 2855	1:1,000	1:2,000
FIS1	Proteintech, No. 10956	1:1,000	1:2,000
Heat shock protein 27 (HSP27)	Enzo, ADI-SPA-800-D mouse	1:1,000	1:2,000
Phospho-heat shock protein 27 (pHSP27) (S82)	CST, No. 2406	1:1,000	1:2,000
Heat shock protein 60 (HSP60) (D6F1) XP(R)	CST, No. 12165	1:1,000	1:2,000
Heat shock protein 70 (HSP70)	Novus, NB110-96427 mouse	1:1,000 (5% milk)	1:2,000
Heat shock protein 90 (HSP90)	CST, No. 4877	1:1,000	1:2,000
IRE1α (14C10)	CST, No. 3294	1:1,000	1:2,000
Phospho-IRE1α (S724)	Abcam, ab48187	1:1,000	1:2,000
Mitofusion-1	Proteintech, No. 66776 mouse	1:1,000	1:2,000
Mitofusion-2	Santa Cruz Technologies, sc-515647 mouse	1:500	1:1,000
mTOR (7C10)	CST, No. 2983	1:500	1:1,000
Phospho-mTor	Abcam, ab109268	1:1,000	1:2,000
Microtubule-associate protein light chain (LC3 A/B)	CST, No. 12741	1:500 (2.5% milk)	1:1,000
SQSTM1/p62	Abcam, ab109012	1:500	1:1,000
Parkin	CST, No. 2132	1:1,000	1:2,000
Phospho-dynamin‐related protein 1 (DRP1) (D6C7)	CST, No. 8570	1:1,000	1:2,000
Protein kinase B (AKT)	CST, No. 9272	1:1,000	1:2,000
Phospho-AKT (S473) (D9E)	CST, No. 4060	1:1,000	1:2,000
Protein kinase-like endoplasmic reticulum kinase (PERK)	Proteintech, No. 24390	1:1,000	1:2,000
Phospho-PERK (Ser719)	Proteintech, No. 29546	1:1,000	1:2,000
PTEN-induced kinase 1 (PINK1)	CST, No. 6946	1:1,000	1:2,000
Phosphoinositide-dependent protein kinase 1 (PDK1) (D37A7)	CST, No. 5662	1:1,000	1:2,000
Phospho-PDK1 (S241) (C49H2)	CST, No. 3438	1:1,000	1:2,000
OPA1	Abcam, ab157457	1:1,000	1:2,000
Ribosomal protein S6 kinase (p70S6K) (4907)	CST, No. 2708	1:1,000	1:2,000
Phospho-p70S6K (T389) (10802)	CST, No. 9234	1:500	1:1,000
ULK1 (D8H5)	CST, No. 8054	1:1,000	1:2,000
Phospho-ULK1 (Ser555) (D1H4)	CST, No. 5869	1:500	1:2,000
XBP-1S (E9V3E)	CST, No. 40435	1:500	1:1,000

### Lyophilized Samples

To assess tissue water content, ∼10-mg samples were lyophilized (Labconco 70181 18 L-50 Series, Kansas City) as previously described (29) and weights were recorded every 24 h until a consistent measure was found, which occurred at 24 and 48 h.

### Histology

Hearts were collected such that the superior 1/3 was fixed in 4% paraformaldehyde for 24 h, after which samples were stored in 70% ethanol. Fixed tissues were paraffin embedded, and 4-µm-thick cross sections were cut and mounted on a slide such that the left ventricle, septum, and right ventricle were visible in each section. Slides were stained with hematoxylin and eosin stain according to standard techniques. Sections were imaged and subjectively scored for injury by a blinded, board-certified veterinary anatomic pathologist. For the subjective evaluation, the entirety of the myocardium was viewed. Lesions were assigned a score of 1 = absent; 2 = mild, focal; 3 = moderate, multifocal; or 4 = marked, widely distributed.

### Statistics

Western blot data were compared using an unpaired two-tailed *t* test with GraphPad Prism 9.3.0 statistical software. Data are presented as means ± SD. Colchicine- and noncolchicine-treated groups were analyzed using the proc MIXED procedure in SAS version 9.4 (SAS Inst., Cary, NC). The model consisted of main effects of environment and colchicine treatment, and data are presented as means ± SD. In our initial consideration of measures, data points two SD away from the mean were identified as outliers and removed from the dataset before analyses regardless of group or directions and are noted in figure legends where relevant. Significance was established as *P* < 0.05.

## RESULTS

**Figure 1.**
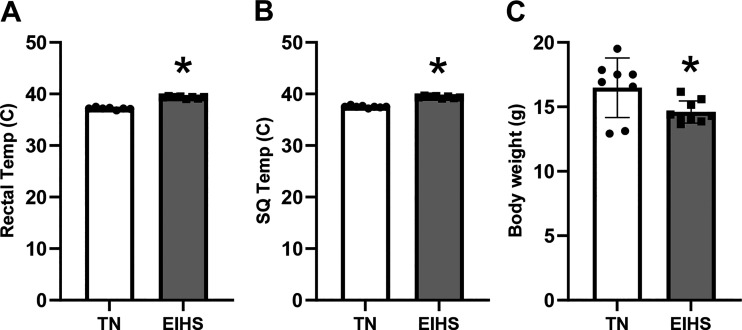
Physiological consequences of environment-induced heat stress (EIHS). EIHS increased rectal temperature (*A*) and SQ temperature (*B*) compared with thermoneutral (TN) and decreased body weight (*C*) compared with TN. Results are expressed as means ± SD. *P < 0.05. TN = 8/group; EIHS = 9/group.

**Figure 2.**
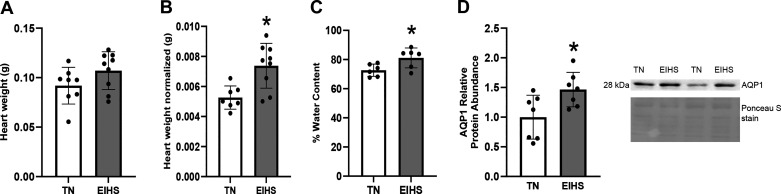
Environment-induced heat stress (EIHS) caused heart architecture to change. Heart weight was similar (*A*); however, EIHS increased heart weight normalized to body weight (*B*), percent water content (*C*), and relative protein abundance of AQP1 (*D*) compared with thermoneutral (TN). Results are expressed as means ± SD. *P < 0.05. *A*: TN = 8/group; EIHS = 9/group. *B*: TN = 8/group, 1 outlier removed; EIHS = 9/group. *C*: TN = 6/group; EIHS = 6/group (limited samples). *D*: TN = 7/group; EIHS = 7/group.

**Figure 3.**
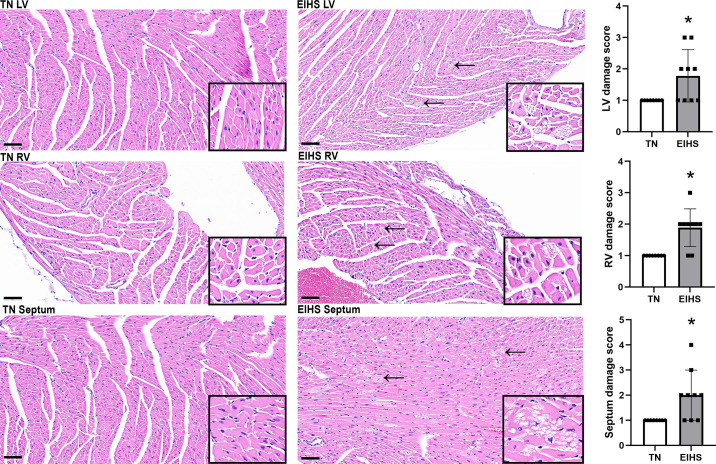
Histological evaluation of the heart following environment-induced heat stress (EIHS). Representative ×40 images from left ventricular (LV), right ventricular (RV), and septum treated with or without EIHS. A trained, blinded pathologist performed a subjective evaluation of degenerative changes in the LV, RV, and septum. For the subjective evaluation, the entirety of the myocardium was viewed. Lesions were assigned a score of 1 = absent; 2 = mild, focal; 3 = moderate, multifocal; or 4 = marked, widely distributed. In LV, RV, and septum, histological inspection suggested increased vacuolation following 24 h of EIHS compared with thermoneutral (TN). Scale bar is 60 µm. Black arrows indicate vacuolation. Inset: images digitally magnified at 250%. *P < 0.05.

To confirm EIHS, RT and SqT were recorded during the environmental challenge. The EIHS treatment increased RT by 2.1°C (*P* < 0.001) and increased SqT by 1.8°C (*P* < 0.001) compared with TN. The final body weight of animals subjected to EIHS was decreased by 10% (*P* = 0.03) compared with TN (Fig. 1). Absolute heart weight was similar between groups, despite the reduction in body weight, and relative heart weight was increased by 26% (*P* < 0.01) in EIHS compared with TN (Fig. 2). Given that the duration of an environmental challenge was unlikely to cause profound cardiac hypertrophy and the large increase in heart weight, we measured tissue water content and relative protein abundance of aquaporin 1 (AQP1), which is frequently associated with edema (34–36). We discovered that water content was increased by 11% (*P* = 0.02) and AQP1 was increased by 46% (*P* = 0.02) in hearts from EIHS animals compared with TN (Fig. 2). To determine the extent to which EIHS may damage the myocardium, we next performed a histological inspection of the left ventricle (LV), right ventricle (RV), and septum. We discovered that EIHS increased myocyte vacuolation in LV (*P* = 0.01), RV (*P* < 0.01), and septum (*P* = 0.01) compared with TN animals (Fig. 3).

**Figure 4.**
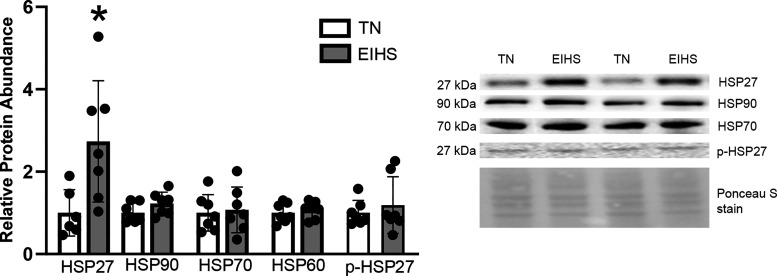
The effect of environment-induced heat stress (EIHS) on heat shock proteins (HSPs). HSP27 was increased by EIHS compared with thermoneutral (TN); however, HSPs 90, 70, 60, and phospho (p)-HSP27 were similar between groups. Results are expressed as means ± SD. *P < 0.05; *n* = 7/group; 1 outlier was removed from TN HSP27.

Given these changes in cardiac histopathology, we next considered the extent to which EIHS may alter cell signaling. Heat shock proteins (HSPs) are known to protect cells from various stressors, including thermic injury. To assess the response to EIHS, we measured relative protein abundance of HSPs 90, 60, 70, 27, and phosphorylated (p)-HSP27 (S82). We discovered that relative protein abundance of HSP27 was increased by 84% (*P* = 0.01) by EIHS compared with TN; however, all other assessed HSPs were similar between groups (Fig. 4).

**Figure 5.**
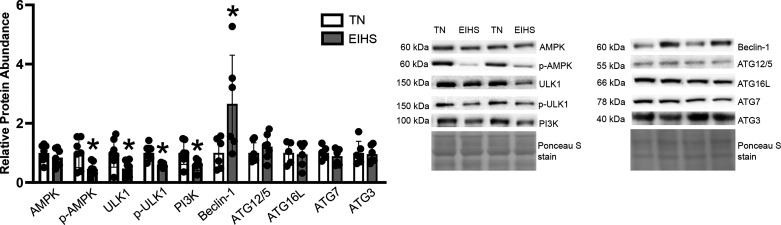
Effects of environment-induced heat stress (EIHS) on autophagy signaling. Relative protein abundance of phospho (p)-AMP-activated kinase (AMPK), UNC-51-like kinase 1 (ULK1), and p-ULK1 were decreased by EIHS compared with thermoneutral (TN), and EIHS increased Beclin-1 compared with TN. AMPK, autophagy-related gene (ATG)12/5, ATG16L, ATG7, and ATG3 were similar between groups. Results are expressed as means ± SD. *P < 0.05. Based on gel size, *n* = 6/group or *n* = 7–9/group; 1 outlier was removed from TN ATG7 and ATG16L, and EIHS phosphatidylinositol 3-kinase class III (PI3K).

To assess the effect of EIHS on autophagy in cardiac muscle, we measured relative protein abundance of proteins that regulate autophagic signaling. Relative protein abundance of total AMP-activated kinase (AMPKα) was similar between groups; however, p-AMPKα (T172) (40H9) decreased by 54% (*P* = 0.02) in EIHS compared with TN (Fig. 5). Total UNC-51-like kinase 1 (ULK1) and p-ULK1 (Ser555) were decreased by 52% (*P* = 0.02) and 40% (*P* < 0.01), respectively, by EIHS compared with TN (Fig. 5). In addition, we measured markers of autophagosome nucleation and elongation. Environment-induced heat stress decreased relative protein abundance of phosphatidylinositol 3-kinases kinase class III (PI3K) by 31% (*P* = 0.04) and increased Beclin-1 by 165% (*P* = 0.04) compared with TN, whereas autophagy-related gene (ATG)12/5, ATG16L, ATG7, and ATG3 were similar between groups (Fig. 5).

**Figure 6.**
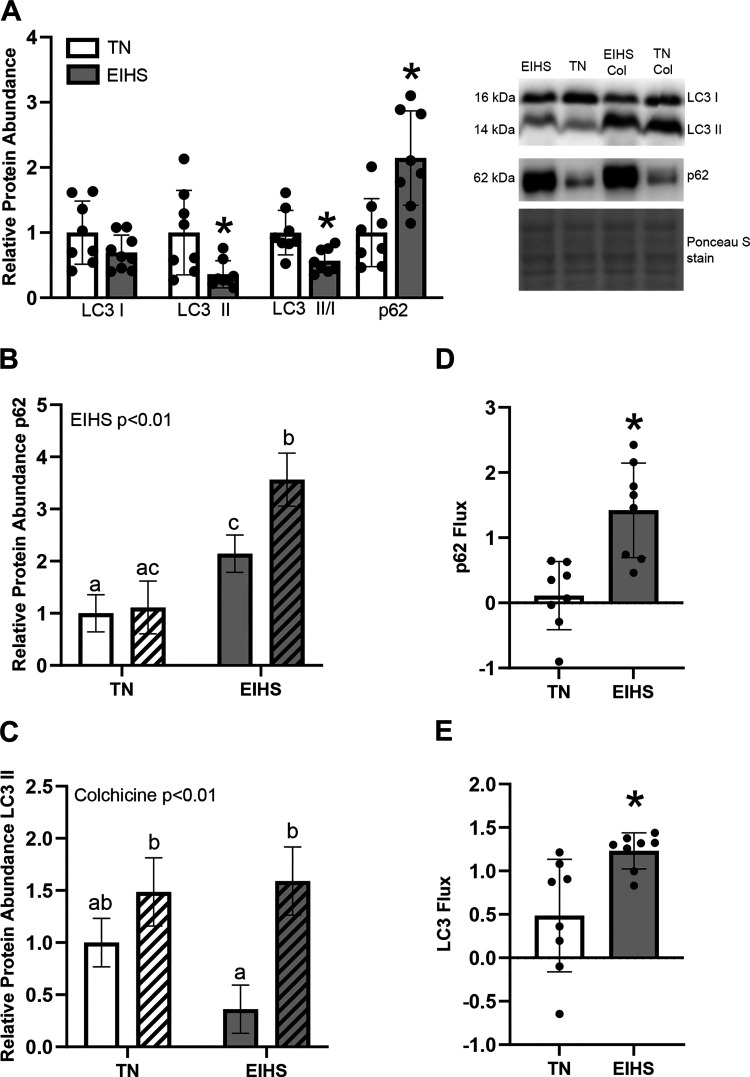
Environment-induced heat stress (EIHS) altered autophagic flux in the heart. *A*: EIHS decreased relative protein abundance of microtubule-associated protein light chain 3 A/B (LC3A/B) II and LC3A/B II/I and increased sequestosome 1 (SQSTM1)/p62 (p62) compared with thermoneutral (TN); however, LC3A/B I was similar between groups. Results are expressed as means ± SD. *P < 0.05; *n* = 8 or 9 per group. *B*: subset of mice was treated with colchicine to allow calculation of flux (*n* = 4/group). There was a main effect of EIHS on relative protein abundance of p62, and colchicine increased relative protein abundance of p62 in EIHS colchicine animals compared with EIHS control animals. *C*: when considering LC3A/B II accumulation caused by colchicine, there was a main effect of colchicine and relative protein abundance of LC3A/B II was increased in EIHS colchicine animals compared with EIHS control animals. In *B* and *C*, different letters represent significant differences (*P* < 0.05). *D* and *E*: EIHS increased flux as assessed by p62 (*D*) and LC3-II (*E*). *B–E*: means ± SD. TN white striped bars, TN colchicine-treated animals; EIHS gray-striped bars, EIHS colchicine-treated animals. *P < 0.05; 1 outlier was removed from EIHS p62, LC3A/B II, and LC3A/B II/I.

To appreciate the extent to which EIHS altered autophagic flux in the heart, a subset of mice was treated with colchicine (37, 38). We discovered that in the EIHS group, microtubule-associated protein light chain 3 A/B (LC3 A/B) I was similar between groups, but LC3 A/B II was decreased by 52% (*P* = 0.01), and the ratio of LC3 A/B II/I was decreased by 34% (*P* < 0.01). We also discovered that EIHS increased sequestosome 1 (SQSTM1)/p62 (p62) by 114% (*P* < 0.01) compared with TN (Fig. 6*A*). We discovered a main effect of EIHS on relative protein abundance of p62 (*P* < 0.01) and colchicine increased relative protein abundance of p62 in EIHS by 30% (*P* = 0.03) compared with EIHS control animals (Fig. 6*B*). When we considered LC3 A/B II accumulation caused by colchicine, we discovered a main effect of colchicine (*P* < 0.01) and that relative protein abundance of LC3 A/B II was increased in EIHS colchicine hearts by 236% (*P* < 0.01) compared with EIHS control hearts (Fig. 6*C*). The accumulation of LC3-II and p62 due to colchicine allows the calculation of flux. Environment-induced heat stress increased flux as assessed by p62 (*P* < 0.01; Fig. 6*D*) and LC3-II (*P* < 0.01; Fig. 6*E*).

**Figure 7.**
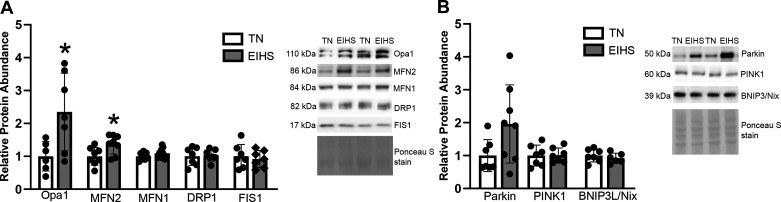
The effects of environment-induced heat stress (EIHS) on mitochondrial fusion/fission and mitophagy proteins. Optic atrophy 1 (Opa1) and mitofusion-2 (MFN2) were increased after 24 h of EHIS compared with thermoneutral (TN; *A*); whereas MFN1, XCC dynamic-related protein 1 (DRP1) and fission protein 1 (FIS1) were similar between groups. Mitophagy proteins were similar between groups (*B*). Results are expressed as means ± SD. *P < 0.05. Based on gel size, *n* = 7/group or *n* = 8 or 9/group. Outlier was removed from TN Opa1 and MFN1 and EIHS MFN2, parkin, and BCL2-interacting protein (BNIP).

Given previous reports of EIHS-mediated mitochondrial injury in swine hearts (29), we also considered the extent to which EIHS would alter mitophagy. We discovered that EIHS increased fusion proteins optic atrophy 1 (OPA1) by 134% (*P* = 0.02) and mitofusion-2 (MFN2) by 65% (*P* = 0.02), though mitofusion-1 (MFN1) was similar between groups (Fig. 7*A*). Environment-induced HS did not appear to promote mitochondrial fission, as relative protein abundance of fission proteins, dynamic-related protein 1 (DRP1) and fission protein 1 (FIS1), was similar between groups (Fig. 7*A*). Finally, mitophagy markers parkin, PTEN-induced kinase 1 (PINK1), and BCL2/adenovirus E1B 19-kDa protein-interacting protein 3-like (BNIP3L/Nix) were similar between groups (Fig. 7*B*).

**Figure 8.**
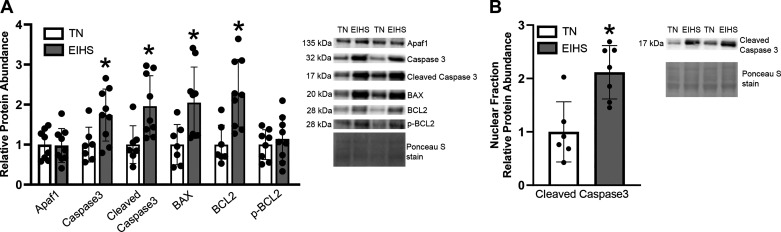
Environment-induced heat stress (EIHS) induced apoptosis in the murine heart. *A*: caspase 3, cleaved caspase 3, BAX, and B-cell lymphoma 2 (BCL2) were increased after 24 h of EIHS compared with thermoneutral (TN), whereas apoptotic protease activating factor 1 (Apaf1) and phospho (p)-BCL2 were similar between groups. *B*: in a nuclear fraction, cleaved caspase 3 was increased after 24 h of EIHS compared with TN. Results are expressed as means ± SD. *P < 0.05. *A*: *n* = 8 or 9/group; 1 outlier was removed from TN caspase 3, cleaved caspase 3, BAX, and BCL2. *B*: *n* = 7/group; 1 outlier removed from TN cleaved caspase 3.

Given the changes in autophagy and mitophagy, we next considered the extent to which EIHS may stimulate apoptosis. We discovered EIHS increased relative protein abundance of caspase 3 by 73% (*P* = 0.02), cleaved caspase 3 by 95% (*P* = 0.01), Bcl-2-associated protein X (BAX) by 1.04-fold (*P* = 0.01), and B-cell lymphoma 2 (BCL2) by 1.2-fold (*P* < 0.01) compared with TN (Fig. 8*A*). However, apoptotic protease activating factor 1 (Apaf1) and p-BCL2 were similar between groups (Fig. 8*A*). We further measured cleaved caspase in nuclear fractions and discovered it increased by EIHS by 1.1-fold (*P* < 0.01) compared with TN (Fig. 8*B*).

**Figure 9.**
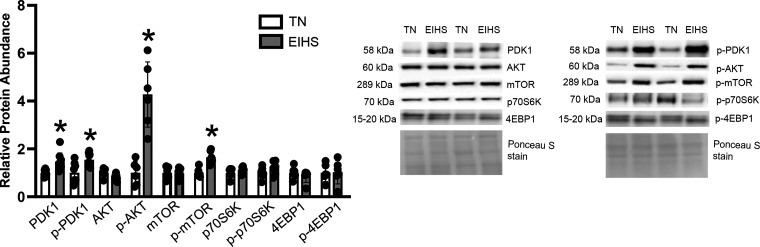
Effect of environment-induced heat stress (EIHS) on mammalian target of rapamycin (mTOR) signaling. EIHS increased phosphoinositide-dependent protein kinase 1 (PDK1), phospho (p)-PDK1, p-AKT, and p-mTOR compared with thermoneutral (TN). Total AKT, total mTOR, and total p70 S6 kinase (p70S6K), phosphorylated p70S6K, total initiation factor 4E binding protein (4EBP1) and p-4EBP1 were similar between groups. Results are expressed as means ± SD. *P < 0.05; *n* = 8 or 9/group for PDK1 and p-PDK1. All other measures used smaller gels such that *n* = 6/group. One outlier was removed from TN PKD1.

Given the role of the mammalian target of rapamycin (mTOR) as a regulator of the stress response and metabolism, we next considered the extent to which EIHS altered mTOR signaling. Herein, we discovered EIHS increased the upstream protein kinase B (AKT) activator, phosphoinositide-dependent protein kinase 1 (PDK1) by 47% (*P* < 0.01) and p-PDK1 (S241) by 54% (*P* < 0.01) and, consistent with these measures, EIHS increased p-AKT (5473) (D9E) by 327% (*P* < 0.01) compared with TN, while total AKT was similar between groups (Fig. 9). Phosphorylated mTOR (S2448) was increased by EIHS by 65% (*P* < 0.01) compared with TN, though total p70 S6 kinase (p70S6K) and p-p70S6K (Thr389) and total initiation factor 4E binding protein (4EBP1) and p-4EBP1 (Thr37/46) were similar between groups (Fig. 9).

**Figure 10.**
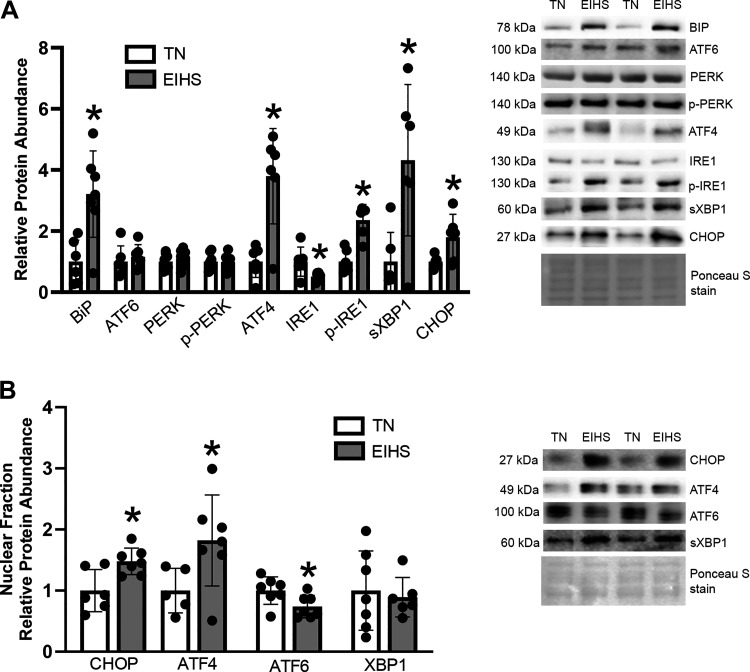
Environment-induced heat stress (EIHS) increased endoplasmic reticulum (ER) stress in the heart. *A*: EIHS increased BiP, activating transcription factor 4 (ATF4), phospho (p)-inositol-requiring enzyme 1α (IRE1α), spliced X-box binding protein 1 (sXBP1), and C/EBP homologous protein (CHOP) compared with thermoneutral (TN) and decreased total IRE1α compared with TN. Total protein kinase-like endoplasmic reticulum kinase (PERK), p-PERK, and activating transcription factor 6 (ATF6) were similar between groups. *B*: in nuclear fraction, EIHS increased CHOP and AFT4 compared with the TN and decreased AFT6; XBP1 was similar between groups. Results are expressed as means ± SD. *P < 0.05. Based on gel size, *n* = 6/group or *n* = 7–9/group. One outlier was removed from TN BiP and CHOP and in nuclear fractions, 1 outlier was removed from TN ATF4.

Given the likelihood of unfolded proteins in response to thermic injury, we measured markers of the unfolded protein response (UPR). In hearts from animals subjected to EIHS, BiP/GRP78 was increased by 220% (*P* < 0.01) compared with TN (Fig. 10*A*). Relative protein abundance of activating transcription factor 6 (ATF6), protein kinase-like endoplasmic reticulum kinase (PERK), and p-PERK (Ser719) were similar between groups, and relative protein abundance of the downstream effector, activating transcription factor 4 (ATF4), was increased by 279% (*P* < 0.01) (Fig. 10*A*). The downstream effectors of inositol-requiring enzyme 1α (IRE1α) are p-IRE1α and spliced X-box binding protein 1 (sXBP1) (39, 40). In hearts from EIHS animals, relative protein abundance of IRE1α was decreased by 48% (*P* = 0.03) but p-IRE1α (S724) was increased by 135% (*P* < 0.01) and sXBP1 was increased by 331% (*P* = 0.01) compared with TN animals (Fig. 10*A*). Finally, EIHS increased C/EBP homologous protein (CHOP) by 79% (*P* = 0.04) compared with TN (Fig. 9*A*). In nuclear fractions, we discovered EIHS increased CHOP by 47% (*P* = 0.01) and ATF4 by 82% (*P* = 0.04) compared with TN animals (Fig. 10*B*), but decreased ATF6 by 26% (*P* = 0.03) and sXBP1 was similar between groups (Fig. 10*B*).

## DISCUSSION

Prolonged exposure to high environmental temperatures can result in a multisystemic stress and jeopardize human health (41). Although this is an existing threat, it is likely to expand because of increased intensity, duration, and frequency of heat waves around the world (42–45). Although others have reported heat stroke-mediated cardiac injury (12, 17, 46, 47), the effects of thermic injury caused by EIHS on cardiac muscle are largely unknown, though EIHS appears to increase the lasting risk of cardiovascular disease (17). The necessity of limited duration and intensity during human experimentation modeling EIHS prevents accurate recapitulation of natural heating emergencies. The purpose of this investigation was to determine the extent to which EIHS alters cardiac health. Given our previous findings, we hypothesized that EIHS would cause myocardial injury and cellular dysfunction.

Our EIHS conditions increased RT and SqT, as well as decreased BW, which is consistent with our previous work using a porcine EIHS model (29, 31). Given the previous findings from our pig model (29), we expected heart weight to decrease and were surprised to discover EIHS increased heart weight in this murine model. Because of the magnitude of the change in heart weight and the relative speed with which it occurred, we reasoned that increased heart weight was driven by edema, which we confirmed. It is clear that there is an increased risk of injury to the heart following heat stroke (8, 9, 48); however, we are not aware of previous reports that have demonstrated shifts in cardiac water content in heat stroke models. Indeed, such a finding was unexpected in this EIHS model, particularly considering shunting of blood to the periphery to support cooling (49). Although a causative mechanism of edema in the heart is not apparent in this model, it, combined with clear histopathological changes in the myocardium, is demonstrative of EIHS-mediated injury, which may lead to impaired cardiac function. Such changes were discovered after cardiac edema caused by lymphatic obstruction impaired contractility and relaxation in a canine ligation model (50). Histopathologic changes may also be suggestive of underlying cellular dysregulation. In rodents, vacuolar degeneration is a well-established indicator of myocardial dysfunction (51). Vacuoles can be a sequela of abnormal accumulation of glycogen, lipids, or swelling of organelles, including lysosomes, sarcoplasmic reticulum, or mitochondria. Further characterization of myocardial vacuoles induced by EIHS will be necessary but was beyond the scope of this investigation.

Despite persistent heating, we discovered that only HSP27 was increased by EIHS. Although surprising, these outcomes are in good agreement with our previous work in oxidative skeletal muscle and left ventricle from our porcine model (29, 52, 53), despite a more robust response in other organ systems (54, 55). In the data reported herein, the entirety of the mouse myocardium was considered en masse, which may have masked ventricle-specific alterations as in the right ventricle from pigs, some EIHS-mediated changes in relative HSP abundance were noted (29). Finally, given our study design, it is possible that alterations in HSP expression resolved before tissue collection, thus avoiding detection.

In our previous work using a porcine model, we discovered impaired degradation of autophagosomes and altered mitophagy in both skeletal muscle and cardiac muscle using a similar EIHS paradigm (29, 32). Given this, and in light of increased edema and histopathological injury, we expected to discover similar changes in the mouse myocardium. Contrary to these expectations, we discovered decreased LC3A/B II and increased p62 abundance as well as increased flux, despite reductions in upstream autophagy activators, p-AMPKα and p-ULK1. Consideration of canonical pathways does not provide direction toward an apparent mechanism; however, we note increased p-mTOR and that, in addition to inhibition of AMPK (56), it may also promote p62 activation (57) and speculate that it may play a role herein.

Likewise, given our previous findings implicating mitochondrial injury (29, 58) and blunted mitophagy (32), we expected similar outcomes in mouse hearts. We discovered that mitochondrial fission proteins were similar between groups; however, mitofusion proteins were increased in EIHS compared with TN. Given increased autophagic flux, it is reasonable to suggest that these changes are reflective of a stress response. Nevertheless, an alternative interpretation of these data could be that mitophagy is inadequately stimulated and damaged mitochondria are in a hyperfused state, which would allow the preservation of damaged mitochondria. Indeed, such preservation causes cellular dysfunction as well as apoptotic signaling (59, 60) and triggers caspase activation (61), as we report herein. Though a shorter heating period, it was previously reported heat stress increased mitochondria membrane permeability and caused activation of caspase-3 through the release of cytochrome-*c* (62). Moreover, we have previously reported EIHS-mediated apoptosis in skeletal muscle, though this followed a brief heating exposure and appeared transient (58).

In addition to increased autophagic flux, there was robust activation of the UPR in hearts from EIHS animals compared with TN animals. The UPR is commonly activated in response to endoplasmic reticulum (ER) stress and promotes survival (63). These changes may indicate that EIHS is causing proteotoxicity and ER stress and that the UPR is contributing to the preservation of cellular health following 24 h of EIHS in murine hearts. Moreover, ER stress is an important inducer of autophagy (63). Although the activation of the UPR may promote stress resistance, chronic activation of the UPR may lead to and be causative of cellular dysfunction and injury (64). The balance of protection and injury caused by the UPR during EIHS will be objectively considered in future investigations.

The intensity, duration, and frequency of environmental heat and subsequent thermic injury is a present and expanding threat to human health. Although translationally relevant, mechanistic investigation using a porcine model is challenging, requiring the development of a murine EIHS model. The outcomes contained herein make clear that EIHS causes injury in mouse hearts, including edema and vacuolation; however, the impact on function is unknown. Furthermore, biochemical outcomes, including increased autophagic flux and UPR are likely reflective of underlying cellular injury. Alternatively, these changes may also contribute to myocardial injury and represent therapeutic targets. How this relationship between cardiac damage and biochemical alterations will develop with longer-term EIHS and/or the return to euthermia will be the focus of future investigation. Furthermore, how age may impact the histological and biochemical consequences of EIHS is also unclear. Herein, we used 8-wk-old mice; however, caution should be taken when extrapolating these outcomes to juvenile mice, fully adult mice, and aged mice (65), particularly as age may mediate the response to and consequences of thermic injury (4, 6). These outcomes also make clear that modifications associated with thermic injury differ between species using similar heating paradigms; a porcine model had biochemical changes reflective of cellular dysfunction, whereas, in this murine model, altered protein expression may support cell survival. Here too, further investigation is necessary as it is important to determine if differences between species are simply driven by the duration of EIHS or if EIHS-mediated changes in pigs and mice are physiologically distinct.

## DATA AVAILABILITY

Data will be made available upon reasonable request.

## GRANTS

This work was supported by U.S. Department of Agriculture Grant 2020-02716.

## DISCLOSURES

No conflicts of interest, financial or otherwise, are declared by the authors.

## AUTHOR CONTRIBUTIONS

M.R. and J.T.S. conceived and designed research; M.R. and J.T.S. performed experiments; M.R., A.M., and J.T.S. analyzed data; M.R. and J.T.S. interpreted results of experiments; M.R. and J.T.S. prepared figures; M.R. and J.T.S. drafted manuscript; M.R., T.E.R., S.K., A.M., and J.T.S. edited and revised manuscript; M.R. and J.T.S. approved final version of manuscript.
